# Critical reflections on the principle of beneficence in biomedicine

**Published:** 2012-02-18

**Authors:** Mawere Munyaradzi

**Affiliations:** 1 University of Cape Town, South Africa; Universidade Pedagogica, Mozambique

**Keywords:** Beneficence, principle, reflections, medical ethics, biomedicine

## Abstract

Medical ethics as a scholarly discipline and a system of moral principles that apply values and judgments to the practice of medicine encompasses its practical application in clinical settings as well as work on its history, philosophy, theology, anthropology and sociology. As such there are a number of values in medical ethics such as autonomy, non-maleficence, confidentiality, dignity, honesty, justice and beneficence, among others. These values act as guidelines for professionals in the medical fraternity and are therefore used to judge different cases in the fraternity. For purposes of this work, this paper examines the principle of beneficence in biomedicine. Using both hypothetical cases and others in real life situations, the paper reflects on the implications of beneficence in biomedicine. It argues that the principle of beneficence is a prima facie obligation that should “always be acted upon unless it conflicts on a particular occasion with an equal or stronger principle”.

## Introduction

Although ethicists have long since discussed values in medical ethics in general, and in particular beneficence, I wish to take issue with it. This is because, in my view, ethicists have not gone far enough in analyzing beneficence, and we still have some way to go towards a comprehensive and systematic approach to the subject (beneficence), especially in terms of its implications and applications to some issues of biomedicine.

In this paper, my argument will proceed in four steps. First, I will shed light on the general understanding of the beneficence as a principle of medical ethics. Second, I will underscore the complexities of beneficence in biomedicine; third, I will consider using case(s), the implications of the principle of beneficence in biomedicine. Finally, I will discuss strategies or possible ways that medical professionals can use the principle of beneficence to benefit both the general public and preserve the integrity of the medical fraternity itself.

## Understanding the nature of the principle of beneficence

The concept of beneficence though widely used in medicine is difficult to define with precision. As such, a number of interpretations have been conjured. However broadly used in English, the word beneficence is considered to mean “the doing of good, the active promotion of good, kindness and charity” [3] or any action that is done for the benefit of others [4]. Though traditionally, acts of beneficence are oftenly done from obligation, the principle is suggestive of altruism, humanity, unconditional love and non-obligatory optional moral ideals. More commonly in medical ethics, beneficence is understood as a principle requiring that physicians provide, and to the best of their ability, positive benefits such as good health, prevent and remove harmful conditions from patients. This is to say that beneficence as a principle of medical ethics asserts an obligation (on the part of the physician) to help others (patients) further their important and legitimate interests and abstain from injuring them in any way, that is, psychologically, morally or physically.

From the foregoing, it can be noted that the central question for beneficence within the patient-physician relationship is: “What does it mean for the physician to seek the greater balance of good over harm in the care of patients?” [5] The beneficence model answers this question at least in terms of the perspective that medicine takes on the patient's best interests rather than the physician's. The model clearly explicates that the central theme for beneficence is the physician's obligation to benefit patients. This has its earliest expression or its primary historical sources in ancient Greece and the Hippocratic Oath which characterizes physicians as a group of committed men (as women were excluded from medicine in the Greek society) set apart from and above others in the society. The central values of the classical Hippocratic ethics were non-maleficence (doing no harm), beneficence and confidentiality. As such, the physician, according to the Hippocratic writings has always had the obligation to “apply dietetic measures to the benefit of the sick according to his ability and judgment, and he ought to keep patients from harms and injustice” [6].

In the modern era, the Hippocratic Oath is traceable to the 18^th^ century with John Gregory and after World War II, medical ethics started to advocate patient autonomy in the guise of informed consent. However, over the last 20 years, there has been growing dissatisfaction with the individual rights-centered ethical framework [7]. Yet like its old version, the Oath stresses on the virtues that keep the physician's attention fixed on his obligations to patients and the latter's best interests, rather than the physician's personal interests. Thus the Hippocratic Oath, by itself, is a “mere” skeleton of the principle of beneficence in so far as it sheds light on concepts that define what it means to be a physician and to “benefit the sick” while avoiding “harm and injustice”’-the moral responsibility of physicians to do away with the sufferings of the sick, and to lessen the violence of their diseases.

## The complexities of beneficence in biomedicine

As has been seen on the nature of the principle of beneficence explicated above, the obligation to confer benefits and actively prevent and remove harms from patients is important in biomedical ethics. However, equally important is the obligation to assess or “weigh and balance the possible goods against the possible harms of an action” [8]. This makes it important to distinguish two principles under the general principle of beneficence-the principle of positive beneficence and the principle of utility [8]. The first principle is known as the principle of positive beneficence. This principle requires the provision of benefits including the prevention and removal of harm from others (i.e. patients). It also includes the promotion of welfare of others. The second version is the principle of utility. This principle, unlike the first, requires weighing and balancing benefits and harms in moral life. This is to say that utility as a principle of beneficence in biomedical ethics makes it imperative for physicians and other health workers to carefully analyze, evaluate and promote those actions that bring more benefits to others (i.e. patients) or the general public.

The second version makes it clear that the principle of beneficence is a prima facie moral obligation. For the moral philosopher, Ross, a prima facie principle is that “principle always to be acted upon unless it conflicts on a particular occasion with an equal or stronger principle” [2]. In other words, a prima facie principle/obligation is that which sometimes is overridden when it conflicts with an equal or a stronger obligation; it is always right and binding, all other things being equal. In the real life situation, we must balance the demands of these principles by determining which carries more weight in the particular case. This is to say that a moral person's “actual” duty is always determined by weighing and carefully balancing all competing prima facie duties in any given situation. This means that the principle of beneficence is not absolute as it is not always binding. Yet this is where the complexity of the principle of beneficence begins in biomedicine. If the principle of beneficence is not absolute in biomedicine, it means that beneficence in biomedicine is not only restricted in application to the patient-physician relationship. It also extends to third parties to that relationship in so far as third parties to the patient-physician relationship can be affected, positively or otherwise. This means that while the physician, according to the principle of beneficence, has the obligation to prevent and remove harm from his/her patients the former can also harm third parties if the physician acts exclusively to benefit the patients. To make this clearer, let us consider the following situation:

*“In a particular city, X lives a couple, W and H. The husband P is HIV positive, but for fear of revealing this information to his wife who is negative and pregnant decides to conceal this information to her. Instead, H sought to arrange a family medical Doctor who helps him with medication to prolong his life. ”*

In this case, the third part, W (to the patient, H –physician relationship) is harmed if the family medical Doctor act exclusively to the benefit of his patient by concealing this information to W. This situation puts the Doctor in a very difficult position especially considering the right of patience to confidentiality. However, the principle of beneficence should be given priority over the principle of respect for patient confidentiality; we need to move beyond individual rights to common good. This is echoed by Margit Sutrop [7] who argues that defense of autonomy and privacy has become an obstacle not only to the use of data in scientific research but also to the use of such information in the implementation of social goals. For him, it has been claimed that epidemiological research is being obstructed, as statistical data cannot be collected without the subject's explicit agreement. Thus coming back to the example given above, respecting third parties will be more desirable. In fact since the principle of beneficence is *prima facie* the second version of the principle-the principle of utility-would require that the third part, W be informed so that she and the foetus are not harmed (not infected as well). By doing so, the Doctor will have removed balanced and removed harms from the third parties (W and the foetus) though H's right to confidentiality will have been violated. Thus in this case, the principle to save more lives (of W and the foetus) is stronger than the right to confidentiality of H. Yet it should be noted and emphasized that the principle of beneficence is always associated with a number of implications especially when used in issues of biomedicine.

## The Implications of beneficence in biomedicine

From the exposition of the nature and complexities of beneficence in the previous sections, it is sufficient to infer that the principle has a number of implications. As previously highlighted, the first principle under the general principle of beneficence-positive beneficence-imply beneficence even to third parties. Put it in other words, since the moral life does not permit us simply to produce benefits without creating risks, positive beneficence would imply that even the third parties to the relationship between the physician and the patient should be benefited. This, however, often creates ethical quandaries-moral dilemmas difficult to solve. One neat case is the example I have given in the previous section, that of a family medical Doctor who happens to know that one of the partners of his clients, H is HIV positive. The Doctor falls in a dilemma of whether s/he should conceal or disclose the information to the third partner (H's wife).

Second, the principle of utility under the general principle of beneficence implies that the interests of the society as a whole should override the individual interests and rights [3]. This implication if granted, can be interpreted to mean that in the context of medical research, for example, the principle entails that dangerous research on human subjects could be undertaken, and even ought to be undertaken, when the prospects of substantial benefits to society/majority outweighs the danger of the research to the individual. In the light of this analysis, the unconstrained principle would allow, for instance, a bone marrow transplant, which has the possibility of risks of the donor becoming a cripple or even dying, to be undertaken from a societal member to benefit a democratic president of a Republic who is suffering from an end-stage organ failure. This example makes it clear that an unconstrained principle of utility carries danger (especially to the minority, unpopular or disadvantaged) with it since it implies that dangerous and sometimes immoral researches on human subjects “ought” to be undertaken. This is echoed by Gallap Survey who argues that the general principle of beneficence especially that with a version of the principle of utility implies that premature or hastened death of individual donors of cadaver organs done in order to benefit patients is justified [9]. Thus for Survey, the principle of utility shows that the principle would justify hastening death of one patient in order to benefit say five others who would procure a heart, a kidney, a liver, an eye and bone marrow each. This situation that beneficence implies is very problematic. It shows that the principle is prone to abuse. As a matter of consequence, unconstrained principle of beneficence generates a sense of distrust and fear for abuse in donors of cadaver organs as they would always worry that physicians might declare them dead prematurely in order to benefit other patients.

Another implication of beneficence has been cited by Peter Singer. He applies the principle in situations such as poverty. For Singer, since requirements of positive action are grounded in principles of preventing or acting to avoid bad outcomes, it implies that “obligatory/over-demanding beneficence requires that we should give until we reach a level at which by giving more, we would cause as much suffering to ourselves as we would relieve through our gift” [10]. Put it differently, positive beneficence implies that we are morally obligated to make large sacrifices and substantially reduce our standard of living in an effort to rescue destitute or poor people around the world. The rich for example would be obliged to reduce their wealth to approximately the level of the poorest person in the world. In medical quarters, the health persons will be obliged to sacrifice their health in order to ameliorate the sick's situations. Thus, though the principle of beneficence is important some of the implications that arise especially in the medical fraternity and other spheres as a result of its presence makes it problematic such that its use and application should be done with caution. The next section makes a critical look at how the principle (of beneficence) should be applied in biomedicine.

## How to apply the principle of beneficence in biomedicine? The way forward

It is a truism that it is hard enough to resolve rationally the moral questions that arise in many cases of biomedicine. One would even think it's a waste of time to pursue such questions. To this kind of thinking, I disagree. I feel obliged to say that moral questions in biomedicine, as in other situations, are not everyone's taste. This is because in my view, moral curiosity and quest for understanding the good and the bad, the right and the wrong are a worthy and even sometimes a noble human characteristic. This is echoed by David Hume who correctly observed that: “It is almost impossible for the mind of man to rest, like those of beasts, in that narrow circle of objects, which are the subject of daily conservation and action” [11]. When we venture of such a narrow circle, we unavoidably bump into questions of moral/ethical nature; human beings can hardly eschew making some judgments about themselves, other human beings and the world. This exercise of making judgment is the beginning of moral reasoning that extends into all spheres of life, biomedicine included.

Though acknowledging that the application of beneficence in most of the issues of biomedicine arguably cause consternation between professionals, patients and members of the public, this does not mean that we should not make judgment of the issues. This is because making judgments and shedding light (through critical questioning) on medical issues help professionals in the medical fraternity to deliberate with ease on some of the difficult issues of biomedicine.

In light of the foregoing, it is argued in this paper that while the principle of beneficence is fundamentally important in the preservation of life, in maximizing patients’ well being, in cost avoidance and risk reduction, the principle like other ethical principles is only fine in theory, but putting it into practice is more difficult as every situation is different [12]. For this reason, I argue that the principle of beneficence is a *prima facie* obligation that should always be acted upon unless it conflicts on a particular occasion with an equal or stronger principle. This entails that the principle should not be universally applied at all times to all cases of biomedicine. Furthermore, the directly involved parties (like patient, patient's relatives, physicians) together with other members such as academics, moralists, representatives of independent organizations, among others should actively take part in the deliberations of controversial issues or cases that arise in biomedicine. Put it in other words, cases of biomedicine should be deliberated by different parties (other than medical professionals and/or patients alone) and the principle of beneficence applied on a case-by-case basis as circumstances of each case are always unique. Margit Sutrop hammering the same point argues that “although autonomy and beneficence seem at times to be in conflict, there is no reason to see one or the other as dominant” [7]. This is because “both autonomy and beneficence as with other ethical principles are needed, but their specific interdependence depends on the particular situation and on social and political context” [7].

## Conclusion

In this study it has been shown that the principle of beneficence like any other principle of medical ethics is important in the preservation of life, in maximizing patients’ well being, in cost avoidance and risk reduction. However, like many other principles of medical ethics, beneficence, especially because of its implications, being a prima facie obligation and the complexities around it, should not always be applied in a universal manner to all cases of biomedicine. Given this scenario, medical professionals often find themselves in a catch twenty-two situation to the extent that it becomes difficult for them to deliberate on many of biomedicine where beneficence is involved. From this observation, it has been argued that there is need by academics and medical professionals, among others, to keep on reflecting on the principles of medical ethics such as beneficence to determine their applicability to different cases that arise in biomedicine. Further to that, the paper has urged that to avoid public outcries, deliberations of issues in biomedicine should be done by many parties, not only by the medical professionals and/or patients alone. More importantly, it has been emphasized that although the principle of benefice is complex and with some far reaching implications, its importance in biomedicine should not be underestimated. The merit of this study therefore lies in its quest to see to it that practitioners in biomedicine recognize the controversies around such principles as beneficence and collaborate with other parties to deliberate on biomedical issues in ways that uphold the ethical integrity of the medical fraternity and illuminate understanding of their practices.

**About the author:** Munyaradzi Mawere is a PhD candidate at the University of Cape Town, South Africa and a senior lecturer at Universidade Pedagogica, Mozambique.
